# Three-year incidence of pacemaker implantation in patients with atrial fibrillation and sinus node dysfunction receiving ablation versus antiarrhythmic drugs

**DOI:** 10.1007/s10840-024-01790-2

**Published:** 2024-04-18

**Authors:** Nazli Kubra Okumus, Emily P. Zeitler, Abdelmoniem Moustafa, Maximiliano Iglesias, Rahul Khanna, Yiran Rong, Saima Karim

**Affiliations:** 1 Allegheny Health Network, Pittsburgh, PA USA; 2 Dartmouth Health, The Dartmouth Institute, Lebanon, NH USA; 3 University of Toledo, Toledo, OH USA; 4 Franchise Health Economics and Market Access, Johnson & Johnson, Irvine, CA USA; 5 Medical Device Epidemiology and Real-World Data Sciences, Johnson & Johnson, New Brunswick, NJ USA; 6 Heart and Vascular Institute, MetroHealth Medical Center, Case Western Reserve University, 2500 MetroHealth Dr, Cleveland, OH USA

**Keywords:** Sinus node dysfunction, Sick sinus syndrome, Atrial fibrillation, Atrial fibrillation ablation, Pacemaker

## Abstract

**Background:**

Sinus node dysfunction (SND) is commonly seen in patients with atrial fibrillation (AF). The purpose of this study was to compare the incidence of pacemaker implantation among patients with SND and AF treated with catheter ablation (CA) versus anti-arrhythmic drugs (AADs).

**Methods:**

The 2013–2022 Optum Clinformatics database, an administrative claims database for commercially insured individuals in the United States (US), was used for this study. Patients with AF and SND and a history of at least one AAD prescription were identified and classified into CA or AAD cohorts based on subsequent treatment received. Inverse probability treatment weighting was applied to balance socio-demographic and clinical characteristics between the cohorts. Weighted Cox regression modeling was used to evaluate the differential risk of incident permanent pacemaker (PPM) implantation. Sub-analyses were performed by AF type (paroxysmal versus persistent).

**Results:**

A total of 1206 patients in the AAD cohort and 1624 patients in the CA cohort were included. Study cohorts were well-balanced post-weighting. The incidence rate of PPM implantation (per 1000 person–year) was 55.8 for the CA cohort and 117.8 for the AAD cohort. Regression analysis demonstrated that the CA cohort had 42% lower risk of incident PPM implantation than those treated with AADs (hazard ratio [HR], 0.58; 95% CI, 0.46–0.72, *p* < 0.001). CA-treated patients had lower risks of PPM implantation versus AAD-treated patients among those with paroxysmal AF (HR, 0.48; 95% CI, 0.34–0.69, *p* < 0.001) and persistent AF (HR, 0.57; 95% CI, 0.40–0.81, *p* = 0.002).

**Conclusions:**

Patients with AF and SND treated with CA have significantly lower risks of incident PPM implantation compared with those treated with an AAD.

**Graphical Abstract:**

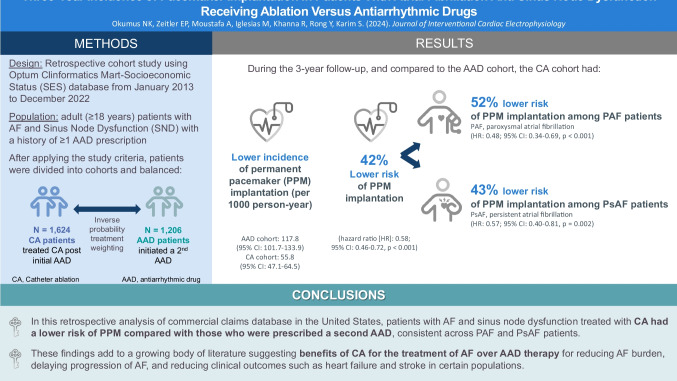

**Supplementary Information:**

The online version contains supplementary material available at 10.1007/s10840-024-01790-2.

## Introduction

Atrial fibrillation (AF) is the most common cardiac arrhythmia among adults affecting up to 6 million people in the US and is expected to rise to 7.2 million by 2035 [1, 2]. Sinus node dysfunction (SND) is characterized by abnormalities in the conductivity and propagation of electrical impulses in the sinoatrial node. AF often coexists with SND with concomitant symptomatic bradycardia, pauses that could lead to symptoms such as fatigue, lightheadedness, syncope, etc.[3] An evaluation by the Outcomes Registry for Better Informed Treatment of Atrial Fibrillation (ORBIT-AF) revealed that 17.7% of patients with AF also had SND at enrollment [4]. AF may be present at the time of initial diagnosis of SND in 40 to 70% of patients [5–7]. SND-induced bradycardia may lead to AF by exacerbating ectopic atrial activity and dispersion of refractorines [8]. Conversely, the sinoatrial node and surrounding atrial tissue may undergo anatomical and electrophysiological remodeling in the setting of AF, which may accelerate the development of SND [9, 10]. The presence of both atrial fibrillation and sinus node dysfunction is also called sick sinus syndrome or tachycardia-bradycardia syndrome.

Catheter ablation (CA) and antiarrhythmic drugs (AADs) are the primary treatment modalities for AF when a rhythm control strategy is pursued. While AADs can help maintain sinus rhythm, they may also affect the function of the sinoatrial node and may worsen preexisting SND [9, 10]. Catheter ablation of AF is a relatively safe procedure that is shown to reduce AF burden, improve quality of life, and even reduce cardiovascular hospitalizations and death, especially in those with heart failure [11–14]. Clinical and observational studies have demonstrated CA to be a more efficacious alternative to AADs in maintaining sinus rhythm in patients with AF [15–19]. Studies have indicated that patients with paroxysmal AF and sinus bradycardia or pauses had impaired progression of sinus node function after ablation, causing longer symptom-free intervals from SND [20–22]. However, there has been limited study of the comparative incidence of permanent pacing among patients with concomitant AF and SND treated with CA versus AADs. While pacemaker insertion is a fairly common procedure, there is a small risk of adverse events in the short and long term, and there are implications on the quality of life as well as a need for future procedures and associated healthcare costs. The purpose of this study was to compare the risk of pacemaker implantation among patients with SND and AF treated with antiarrhythmic drug therapy versus catheter ablation.

## Methods

### Data source

This retrospective cohort study used the Optum Clinformatics Data Mart-Socioeconomic Status (SES) database from January 1, 2013, to December 31, 2022. Optum SES data is an administrative claims database for commercially insured and Medicare Advantage beneficiaries in the United States. The database includes inpatient, outpatient, and pharmacy claims for more than 15 million patients annually. This analysis of the Optum SES data was conducted under an exemption from Institutional Review Board oversight for US-based studies using de-identified healthcare records, as dictated by Title 45 Code of the Federal Regulations (45 CFR 46.101(b)(4)) (https://www.govinfo.gov/content/pkg/CFR-2011-title45-vol1/pdf/CFR-2011-title45-vol1.pdf).

### Study population

This study included adults (aged ≥ 18 years) with AF (*International Classification of Diseases*, Ninth Revision, Clinical Modification [ICD-9-CM] diagnosis codes: 427.31; *International Classification of Diseases*, Tenth Revision, Clinical Modification [ICD-10-CM] diagnosis codes: I48.0, I48.1x, I48.2x, I48.91). For inclusion, these patients had to have a prior history of being prescribed at least one AAD (having at least a 30-day supply fill of medications including amiodarone, dofetilide, dronedarone, flecainide, propafenone, sotalol, disopyramide, and quinidine between January 1, 2014 and December 31, 2022). The criteria for inclusion were established in accordance with the Class I recommendation outlined in the 2023 ACC/AHA/ACCP/HRS guidelines for the diagnosis and management of atrial fibrillation. This recommendation suggests that patients with symptomatic atrial fibrillation for whom antiarrhythmic medications are either not tolerated, not proffered, or contraindicated should undergo catheter ablation (CA) with certain exceptions [23].

Patients were then classified into two cohorts: (1) one cohort received catheter ablation or CA cohort if their subsequent treatment post the initial AAD was CA for AF (with the date of the initial CA procedure considered as the index date for CA cohort), and (2) the second cohort is AAD cohort if they initiated a second AAD (with the date of the first fill of second AAD considered as the index date for AAD cohort). The method for identifying the catheter ablation cohort was based on the Current Procedural Terminology [CPT] code—93651, 93656; the ICD-9 Procedural Coding System [PCS]—37.34; and the ICD-10 PCS—02553ZZ, 02563ZZ, 02573ZZ, 02583ZZ, 025K3ZZ, 025L3ZZ, 025M3ZZ, 025S3ZZ, and 025T3ZZ. Patients were required to have a medical service visit with a primary or secondary diagnosis of SND (ICD-9-CM diagnosis code, 427.81; ICD-10-CM diagnosis code, I49.5) in 12-month period prior to their treatment index date. Patients were also required to be continuously enrolled between the first AAD fill and index date. Continual enrollment was further required in the 12 months before the index date for patients with their first prescription of AAD being within 12 months of their index date. Patients were excluded if they had a prior history of PPM or implantable cardioverter defibrillator in the pre-index AAD period (all available data). Patients were also excluded if they had a prior catheter or surgical ablation, AV nodal ablation, valvular procedure, left atrial appendage occlusion, or high-grade/complete heart block in the 12-month pre-index AAD period.

### Outcomes of interest

The outcome of interest was incident PPM implantation (identified based on CPT codes 33206, 33207, 33208, and 33274; Healthcare Common Procedure Coding System [HCPCS] codes C2619, C2620, C2621, C1785, and C1786; and ICD-9/ICD-10 PCS codes 00.50, 00.53, 37.80, 37.81, 37.82, 37.83, 0JH604Z, 0JH605Z, 0JH606Z, 0JH607Z,0JH634Z, 0JH635Z, 0JH636Z, 0JH637Z, 0JH804Z, 0JH805Z, 0JH806Z, 0JH807Z, 0JH834Z, 0JH835Z, 0JH836Z, and 0JH837Z) in the 3-year follow-up period post-index treatment assignment. Data were censored if a patient had lost to follow-up during the designated period. Patients in the AAD group were also censored if they underwent CA within the 3-year follow-up period.

### Study covariates

Patients’ demographics including age, sex, race/ethnicity, geographic region, education, and household income were reported. Elixhauser comorbidity score [24] and CHADS_2_VASc score [25] were measured based on the medical claims in the 12-month baseline period. Other clinical characteristics that could contribute to worsening sinus node dysfunction including right bundle branch block, left bundle branch block, intraventricular conduction delay, prior cardiac surgery, cardiomyopathy, and sleep apnea were assessed. In addition, prescriptions for beta blockers, calcium channel blockers, digoxin, and oral anticoagulants were included as covariates.

### Statistical analysis

The inverse probability of treatment weighting (IPTW) approach was applied to balance sociodemographic and clinical comorbid characteristics between the study cohorts [26]. Each patient was assigned a weight by using the IPTW technique along with an estimation of the average treatment effect [27]. As a first step, a logistic regression model was performed to estimate the propensity score of an individual to undergo CA, adjusting for covariates. In the next step, the inverse of the propensity scores was used to generate weights for each patient. IPTW weights were stabilized within each cohort to reduce the variance of the estimator. Standardized mean difference (SMD) was used to assess if the distribution of these covariates was balanced after weighting (with absolute SMD > 0.1 considered imbalanced).

Incidence rate (per 1000 person–year) and 3-year cumulative incidence of pacemaker implantation as well as corresponding 95% confidence intervals (CIs) were estimated for both CA and AADs treatment groups. Kaplan–Meier curves were used to visualize the incidence of PPM implantation by treatment groups. Weighted Cox regression modeling was used to evaluate the differential risk of incident PPM. Sub-analyses were performed by AF type (paroxysmal versus persistent AF based on ICD-10 codes [I48.0, and I48.1 and I48.2, respectively, during the index or on the most recent medical visit in the 12 months prior to the index]). To account for the potential effect of AAD use on sinus node function in the CA cohort (post-index CA treatment assignment), a sensitivity analysis was conducted by censoring patients who had an AAD prescription filled in the CA treatment group. All statistical analyses were conducted with R software (version 4.1.2; R Foundation for Statistical Computing, Vienna, Austria).

## Results

### Patient population

**Fig. 1:**
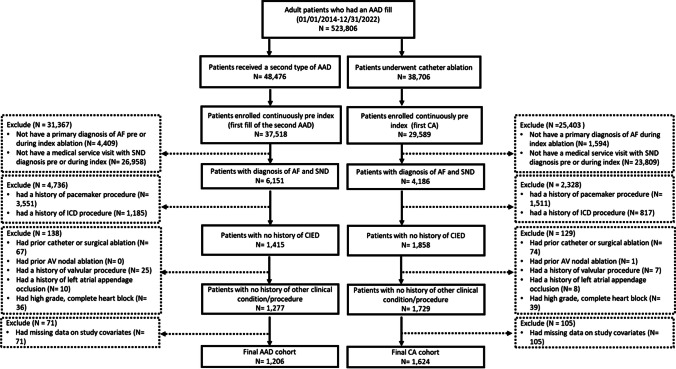
Patient inclusion/exclusion flow chart

A total of 1206 patients in the AAD cohort and 1624 patients in the CA cohort met the study inclusion and exclusion criteria (Fig. 1). In the pre-weighted cohort, patients in the catheter ablation cohort had a lower proportion of those aged ≥ 70 years (53.0% vs. 69.5%, absolute SMD [aSMD] for age = 0.347) and fewer female patients (46.5% vs. 53.5%, aSMD = 0.140) as compared with the antiarrhythmic cohort. Also, the CA cohort had a lower proportion of patients who had CHADS_2_-VASc score ≥ 2 (89.4% vs. 93.8%, aSMD = 0.158) and a higher percentage of patients who had a history of cardiomyopathy (21.1% vs. 13.5%, aSMD = 0.202), sleep apnea (37.1% vs. 29.2%, aSMD = 0.168), and anticoagulant use (87.7% vs. 77.8%, aSMD = 0.264) versus those in the AAD cohort. The proportion of patients with a beta-blocker prescription (79.2% vs. 76.7%, aSMD = 0.062) and calcium channel blocker prescription (24.5% vs. 26.7%, aSMD = 0.050) were similar between the two groups. After weighting, aSMD values suggested a good balance on all study covariates between the CA and AAD cohorts (all < 0.1) (Table 1).

**Table 1:** Patient characteristics by cohort pre- and post-inverse probability of treatment weighting

	Pre-weight cohort			Post-weight cohort		
	AAD (*n* = 1206)	CA (*n* = 1624)	aSMD	AAD	CA	aSMD
Study characteristics	*n* (%)	*n* (%)		%	*n* (%)	
Age (year)			0.347			0.010
18–49	20 (1.7%)	39 (2.4%)		2.2%	2.1%	
50–59	80 (6.6%)	144 (8.9%)		8.1%	7.9%	
60–69	268 (22.2%)	581 (35.8%)		29.8%	29.9%	
≥ 70	838 (69.5%)	860 (53.0%)		60.0%	60.1%	
Gender			0.140			0.007
Female	645 (53.5%)	755 (46.5%)		48.7%	49.1%	
Male	561 (46.5%)	869 (53.5%)		51.3%	50.9%	
Race/ethnicity			0.060			0.009
White	984 (81.6%)	1356 (83.5%)		82.6%	82.4%	
Black	86 (7.1%)	113 (7.0%)		7.0%	7.2%	
Hispanic	82 (6.8%)	97 (6.0%)		6.4%	6.5%	
Other	54 (4.5%)	58 (3.6%)		4.1%	4.0%	
Education			0.132			0.007
≤ High school	332 (27.5%)	366 (22.5%)		24.7%	24.8%	
Attended collage	683 (56.6%)	944 (58.1%)		57.8%	57.5%	
≥ Bachelor’s degree	191 (15.8%)	314 (19.3%)		17.5%	17.7%	
Annual household income ($)			0.173			0.007
< 50 k	458 (38.0%)	496 (30.5%)		33.4%	33.7%	
50–99.9 k	465 (38.6%)	652 (40.1%)		39.6%	39.5%	
≥ 100 k	283 (23.5%)	476 (29.3%)		27.0%	26.8%	
Geographic region			0.061			0.007
Midwest	268 (22.2%)	361 (22.2%)		22.1%	22.0%	
Northeast	87 (7.2%)	122 (7.5%)		7.6%	7.5%	
South	553 (45.9%)	780 (48.0%)		47.2%	47.2%	
West	298 (24.7%)	361 (22.2%)		23.1%	23.3%	
Elixhauser comorbidity index			0.073			0.008
** 0–1**	10 (0.8%)	26 (1.6%)		1.2%	1.3%	
** 2–3**	214 (17.7%)	275 (16.9%)		17.6%	17.3%	
** ≥ 4**	982 (81.4%)	1323 (81.5%)		81.2%	81.4%	
CHA_2_DS_2_-VASc			0.158			0.004
< 2	75 (6.2%)	172 (10.6%)		8.8%	8.7%	
≥ 2	1131 (93.8%)	1452 (89.4%)		91.2%	91.3%	
Comorbidity						
Cardiomyopathy	163 (13.5%)	343 (21.1%)	0.202	18.6%	18.0%	0.013
Right bundle branch block	27 (2.2%)	20 (1.2%)	0.077	1.5%	1.4%	0.010
Left bundle branch block	7 (0.6%)	7 (0.4%)	0.021	0.5%	0.5%	0.003
Intraventricular conduction delay	9 (0.7%)	11 (0.7%)	0.008	0.7%	0.7%	0.001
Sleep apnea	352 (29.2%)	602 (37.1%)	0.168	34.4%	33.9%	0.010
History of cardiac surgery	117 (9.7%)	148 (9.1%)	0.020	9.6%	9.5%	0.003
Use of medication						
Beta blocker	925 (76.7%)	1287 (79.2%)	0.062	78.4%	78.3%	0.002
Calcium channel blocker	322 (26.7%)	398 (24.5%)	0.050	25.2%	25.5%	0.007
Digoxin	101 (8.4%)	151 (9.3%)	0.033	9.0%	9.0%	0.001
Oral anticoagulant	938 (77.8%)	1424 (87.7%)	0.264	83.3%	83.2%	0.003

*AAD* antiarrhythmic drug, *CA* catheter ablation, *aSMD* absolute standardized mean difference

### Risk of PPM implantation

The incidence rate of PPM implantation was 55.8 per 1000 person–year (95% CI, 47.1–64.5 per 1000 person–year) for the CA cohort and 117.8 per 1000 person–year (95% CI, 101.7–133.9 per 1000 person–year) for the AAD cohort (Table 2). The 3-year cumulative incidence of PPM implantation was 13.8% (95% CI, 11.6–15.9%) for the CA cohort versus 23.1% (95% CI, 20.0–26.2%) for the AAD cohort, respectively (Table 2).

**Table 2:** Pacemaker implantation by treatment modalities

Pacemaker implantation	No. of pacemaker/person–year	Incidence rate (per 1000 person–year) and 95% CI	3-year cumulative incidence	HR and 95% CI
AAD	206/1749	117.8 (101.7, 133.9)	23.1% (20.0%, 26.2%)	REF
CA	158/2833	55.8 (47.1, 64.5)	13.8% (11.6%, 15.9%)	**0.58 (0.46, 0.72), *p* < 0.001**

*AAD* antiarrhythmic drug, *CA* catheter ablation, *REF* reference group

**Fig. 2:**
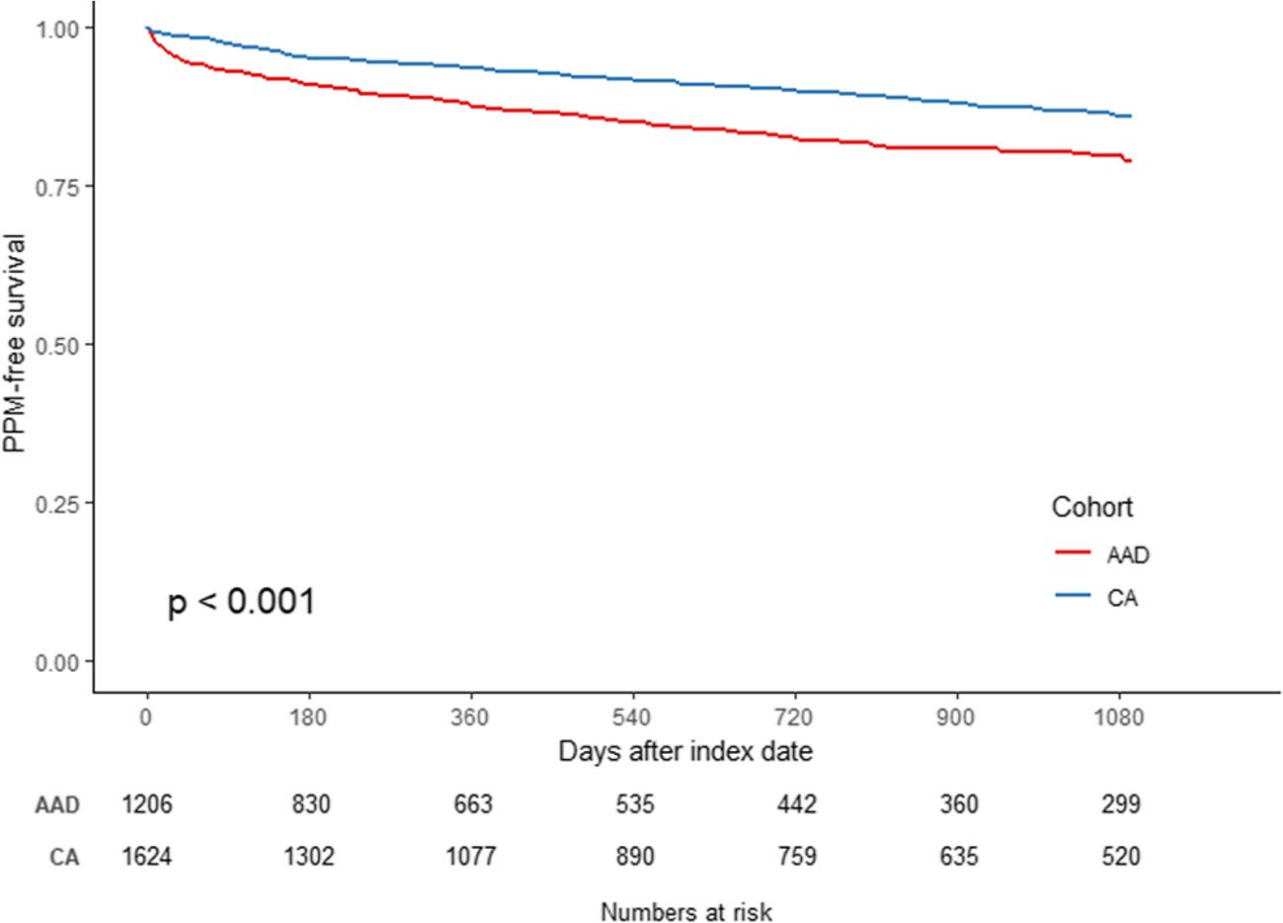
Kaplan–Meier curve of incidence of pacemaker implantation by treatment modalities

**Fig. 3:**
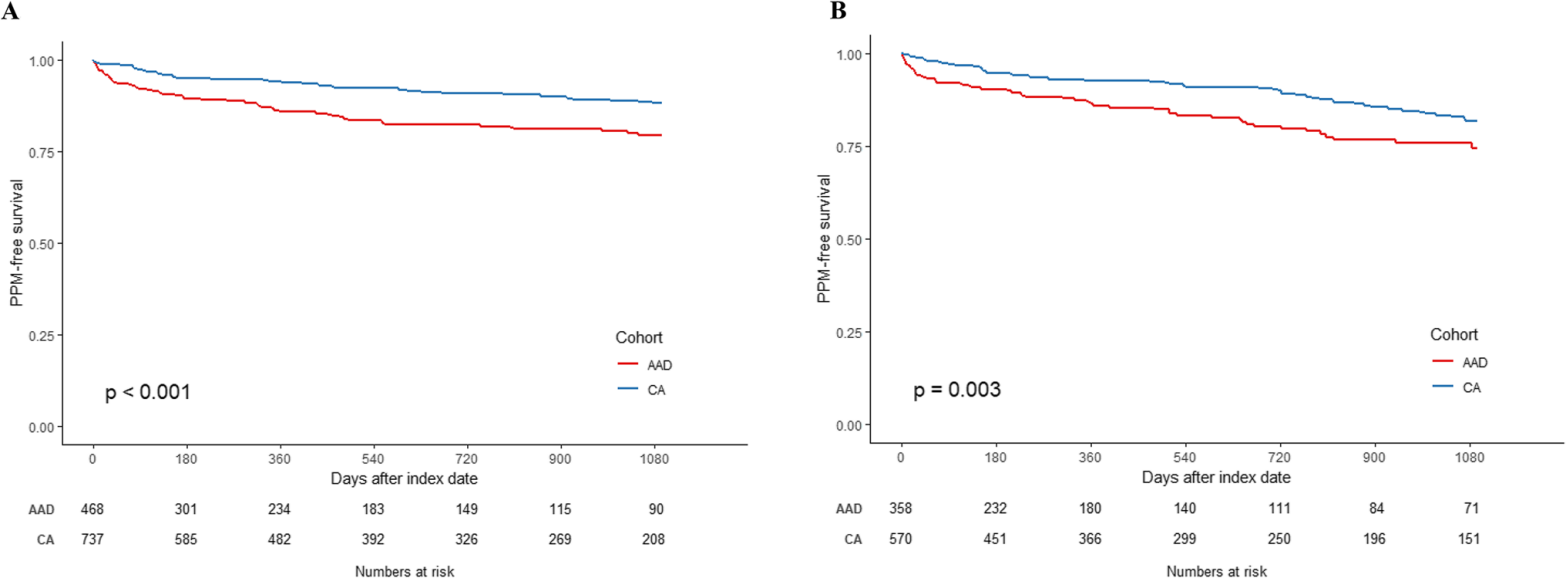
Kaplan–Meier curve of incidence of pacemaker implantation by treatment modalities. **A** Patients with paroxysmal AF; **B** patients with persistent AF

The Kaplan–Meier curve depicts the PPM implantation-free survival during the 3-year follow-up in the two groups post-weighting (Fig. 2) and suggests that the risk of PPM implantation was significantly lower in the CA cohort (log-rank *p*-value < 0.001). A weighted Cox regression model demonstrated that patients treated with CA had a 42% lower risk of incident PPM as compared with those treated with an AAD for AF patients identified using the ICD-9 and ICD-10 codes (hazard ratio [HR], 0.58; 95% CI, 0.46–0.72; *p* < 0.001). Similar results were observed when examined for patients with paroxysmal versus persistent AF when utilizing the ICD-10 codes for the capture of classification of AF (Fig. 3A and B). Among patients with paroxysmal AF, those treated with CA had a 52% lower risk of PPM implantation as compared with those treated with an AAD (HR, 0.48; 95% CI, 0.34–0.69; *p* < 0.001). Similarly, among patients with persistent AF, the risk of PPM implantation was 43% lower for the CA cohort as compared to the AAD cohort (HR, 0.57; 95% CI, 0.40–0.81; *p* = 0.002).

### Sensitivity analysis

**Fig. 4:**
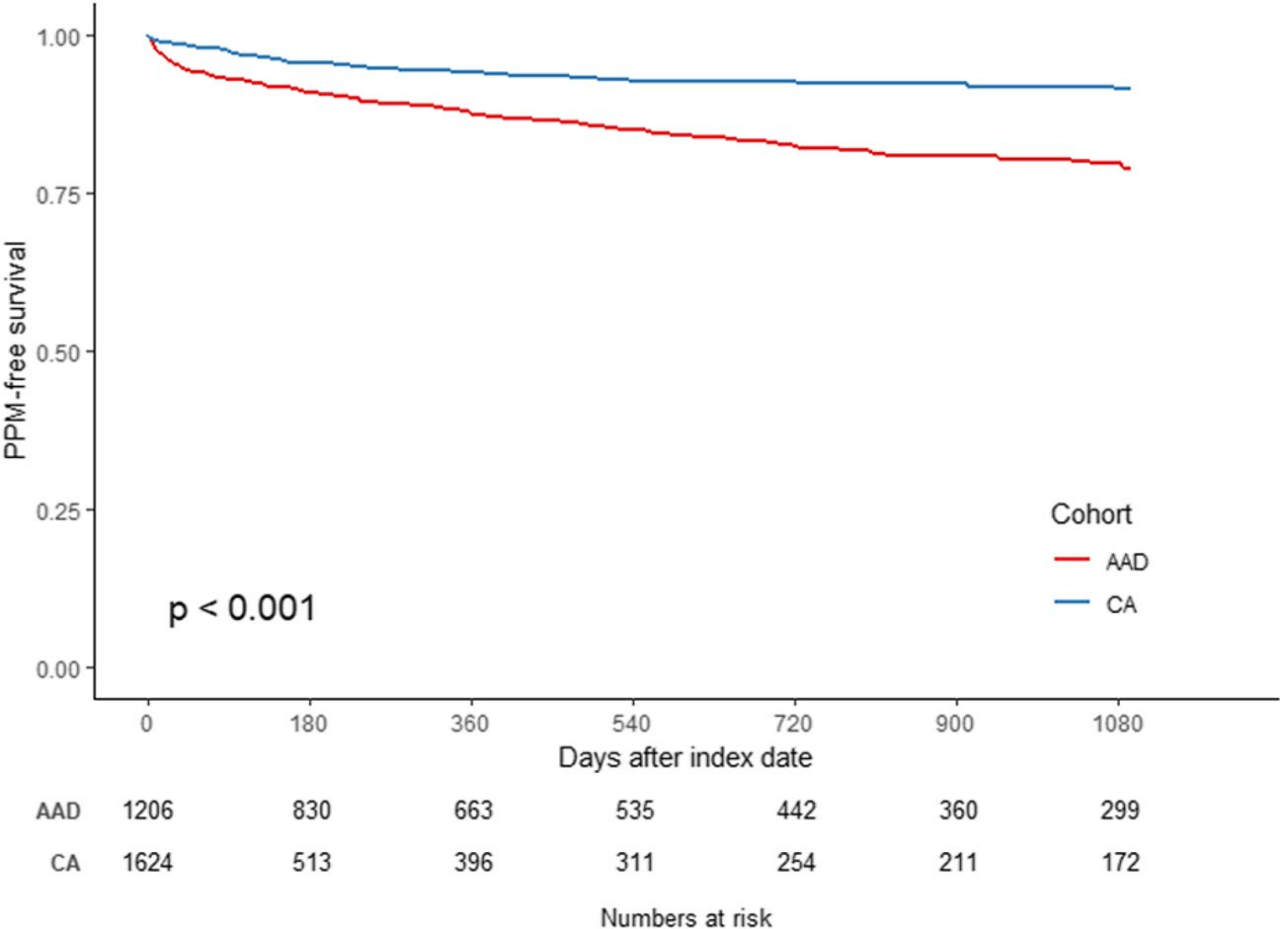
Kaplan–Meier curve of incidence of pacemaker implantation by treatment modalities censoring those prescribed with an AAD after CA

Results from the sensitivity analysis, wherein CA patients were censored if they had a post-CA prescription fill for an AAD, were consistent with those from the main analysis (Fig. 4; Supplementary Appendix Table 1, Fig. 1). The CA cohort had a 60% lower risk of incident PPM as compared with the AAD cohort despite a more rigorous censoring rule being applied (HR, 0.40; 95% CI, 0.29–0.56; *p* < 0.001).

## Discussion

Using a nationally representative administrative claims database, we assessed the difference in incident pacemaker implantation rates among patients with sick sinus syndrome treated with CA versus AADs. In this study, patients treated with CA had a 42% lower risk of incident PPM implantation during the 3-year follow-up, as compared with those treated with an AAD.

Prior studies have suggested that successful ablation of AF among patients with SND can improve or resolve bradyarrhythmia and pauses, thereby preventing or delaying the need for pacing [20–22]. Hocini et al. observed a significant improvement in the sinus node function and no bradycardia or sinus pause-associated symptoms post-radiofrequency ablation of AF among patients with paroxysmal AF and prolonged conversion pauses (≥ 3 s) [20]. In a retrospective study by Khaykin et al. [22], patients with symptomatic sinus bradycardia or pauses were observed to have significantly increased mean heart rate and reduced atrial pacing burden after pulmonary vein isolation for AF. In another study of patients with concomitant paroxysmal AF and tachycardia-bradycardia syndrome, sinus pauses were eliminated in over 90% of patients after AF ablation, and PPM was required in only three patients (8%) during the 5-year follow-up [21]. The results of our study, which included real-world data from a large cohort of patients add to this body of literature and demonstrate that catheter ablation in patients with AF and SND is associated with reduced risk of pacemaker implantation over 3 years compared with the use of antiarrhythmic therapy.

The relationship between catheter ablation and improvement in sinus node function is not fully understood. The reduced need for pacemakers in those with sick sinus syndrome who received catheter ablation may be attributable to electrical reverse remodeling of the sinus node. It has been demonstrated that the improved sinus node function (i.e., increased mean heart rate, maximal heart rate, and heart rate range and decreased corrected sinus node recovery time) can be linked to the elimination of sinoatrial node overdrive suppression and the prolonged sinus pauses by CA [20]. Interestingly, there was a greater reduction in the need for pacemakers in those with paroxysmal atrial fibrillation versus persistent atrial fibrillation, which may be related to the possible extent of reverse remodeling. Sensitivity analysis which censored patients prescribed an AAD post-CA revealed a greater reduction in PPM implantation risk (OR, 0.40 vs. 0.58) for CA-treated patients versus those who were on AADs, potentially reflecting catheter ablation effects on SND despite antiarrhythmic use post-ablation in this population. Other potential explanations are based on the fact that CA, on average, dramatically reduces the burden of AF which has at least two potential effects related to the need for pacing. First, reduced AF burden allows for the elimination or reduction of rate control agents and AADs that suppress the function of the sinoatrial (and atrioventricular) node. Many rate control medications and AADs can worsen preexisting sinus bradycardia or pauses and should be used with caution in patients prone to this condition [9, 10]. Additionally, reducing AF burden—especially among patients with paroxysmal AF—may have the added benefit of reducing conversion pauses which are a common complication of AF leading to PPM implantation [9, 10].

The incremental reduction in risk of pacemaker implantation was consistent for patients with paroxysmal or persistent AF who underwent atrial fibrillation ablation. It has been demonstrated that early ablation of AF can improve outcomes (i.e., lower rate of death, stroke, hospitalization or AF recurrence, and improved quality of life) in patients with paroxysmal atrial fibrillation [15, 28]. Furthermore, Butt et al. [29] demonstrated in a large cohort of patients that earlier ablation of atrial fibrillation after diagnosis of AF and SND was associated with a decreased risk of pacemaker implantation, which may further support the hypothesis of reversible electrical remodeling in a temporal manner. While prior studies have demonstrated a correlation between atrial fibrillation diagnosis to ablation time and the need for pacemakers, the current study looks at atrial fibrillation classification with findings that suggest that patients with both SND and either persistent AF or paroxysmal AF benefit from a lower risk of pacemaker implantation following catheter ablation of AF compared with antiarrhythmic therapy. The temporal nature of the reduction in the need for pacemakers depending on the timing of ablation after diagnosis of atrial fibrillation and SND may explain the further reduction in the need for pacemakers in those with paroxysmal atrial fibrillation compared to persistent atrial fibrillation.

There are several strengths of this study. First, patients with AF and SND are directly compared based on the rhythm control strategy they underwent, which addresses a commonly encountered significant clinical scenario with substantial treatment uncertainty. There will be patients with profound sinus node dysfunction that may not be reversible and may need a pacemaker regardless of AF therapy. However, this study incorporates real-world data from a large number of patients and demonstrates a decreased use of pacemakers if atrial fibrillation ablation is performed compared with AADs as a second-line strategy after initial antiarrhythmic use (consistent with current guidelines for atrial fibrillation ablation) in patients with sinus node dysfunction and atrial fibrillation. Using real-world data also allows for analysis of a more generalized cohort of patients of diverse racial and ethnic backgrounds, sex, and comorbidity status, as compared with populations in clinical trials involving ablation [30].

There are several limitations of this study. The effect of potential confounders that are not identifiable through claim databases, including the severity of SND and AF, was not included in our analysis. While patient-level data, including heart rate before and after CA or the incidence of right-sided ablations, were not available, we did include all available variables that could impact the conduction system including comorbidities such as prior cardiac or valvular surgery and prescriptions for medications that could suppress sinus node function, intraventricular conduction delay, bundle branch block, etc. The role of immortal time bias in influencing study results cannot be ruled out; however, any impact is likely to be minimal as the time gap between the first AAD and catheter ablation for the ablation cohort and the time gap between the first AAD and second AAD for the AAD cohort was not significantly different (mean 482.51 days [standard deviation 548.44 days] vs. 505.03 days [standard deviation 573.06 days], *p*-value = 0.289). In our research, we stratified the patients according to their AF type using the ICD-10 coding system, which can be prone to errors in entry. However, the large number of patients in our cohorts likely overcomes the possibility of these errors or inconsistencies having a clinical impact on the outcomes.

Misclassification of atrial fibrillation can occur if the disease progression occurred during claims processing period from paroxysmal to persistent, and this change could have influenced study results. Additionally, both patient cohorts had already received antiarrhythmic medication as a first-line therapy and were then divided into two cohorts based on subsequent treatment selection. Therefore, the analysis does not include patients who never received antiarrhythmic therapy, so the generalization of our findings to that group is limited. The antiarrhythmic therapy selection as first and second line was as per physician discretion, but the antiarrhythmic selections were the ones that are commonly prescribed. Another limitation is the variation in the definition of SND in clinical practice, which could lead to inconsistencies in clinical practice. However, the large size of our cohort will likely overcome variations in these definitions from having a statistically significant impact on the clinical outcome. Additionally, patients with AF and SND who had already undergone PPM implantation were excluded from the study. Despite this limitation, our cohort of patients with AF and subsequent SND diagnosis with no history of pacemaker implantation remains a substantial proportion of the patient population, as demonstrated by the paper by Butt et al. [29], which included 66,595 patients with AF and SND without previous PPM implantation. Additionally, the presence of AF can coexist with other conduction abnormalities, such as LBBB, which could serve as an indication for cardiac resynchronization therapies in patients with heart failure [31]. Although this could potentially be a confounding factor in such a study, the prevalence of LBBB was 0.4% and 0.6% in the AAD and CA groups, respectively, which is unlikely to have a statistically significant impact on the overall results given the high number of patients included in the study. A prior study by Butt et al. [29] has also shown that earlier catheter ablation for atrial fibrillation mitigated the need for pacemaker implantation compared to antiarrhythmic therapy even when accounting for conduction system disorders that could lead to a higher incidence of pacemaker implantation [29].

Optum database only includes commercially insured patients in the US. As such, our study findings may not be generalizable to uninsured patients or patients with non-commercial insurance.

## Conclusion

Following treatment with an antiarrhythmic drug, patients with atrial fibrillation and sinus node dysfunction treated with catheter ablation had a significant reduction in the incidence of pacemaker implantation compared with those who were prescribed a second antiarrhythmic drug. The reduced need for pacemaker implantation over time associated with the catheter ablation arm was consistent across patients with persistent and paroxysmal atrial fibrillation. The study results suggest that among patients with sick sinus syndrome, catheter ablation significantly decreases the risk of future need for a permanent pacemaker as compared with antiarrhythmic therapy given the potential for improvement of sinus node function as well as decreased need for further therapies that may further contribute to sinus node dysfunction. These findings add to a growing body of literature suggesting the benefits of atrial fibrillation ablation over antiarrhythmic drug therapy for reducing atrial fibrillation burden, delaying the progression of atrial fibrillation, and reducing clinical outcomes such as heart failure and stroke in certain populations.

## Supplementary Information

Below is the link to the electronic supplementary material.

Supplementary file1 (DOCX 13.3 KB)

## Data Availability

Optum Clinformatics data can be availed through purchase/license agreement with Optum©.
