# Supplementary material for: Three-year incidence of pacemaker implantation in patients with atrial fibrillation and sinus node dysfunction receiving ablation versus antiarrhythmic drugs
Source: J Interv Card Electrophysiol. 2024 Apr 18;67(7):1593–602. doi: 10.1007/s10840-024-01790-2 (PMC11522185; doi:10.1007/s10840-024-01790-2)
Supplement: Supplementary file 1 — Supplementary file1 (DOCX 13.3 KB) [file 10840_2024_1790_MOESM1_ESM.docx]

**Supporting Information**

**Table S1. Risk of pacemaker implantation by treatment modalities for sensitivity analysis (censoring those prescribed an AAD after CA)**

|  | **No. Pacemaker/Person-year** | **Incidence rate (per 1,000 person-year) and 95% CI** | **3-year cumulative incidence** | **HR and 95% CI** |
| --- | --- | --- | --- | --- |
| **Pacemaker implantation** |  |  |  |  |
| **AAD** | 206/1,749 | 117.8 (101.7, 133.9) | 23.1% (20.0%, 26.2%) | REF |
| **CA** | 54/1,106 | 48.8 (35.8, 61.8) | 8.3% (5.7%, 10.8%) | 0.40 (0.29, 0.56), p < 0.001 |

Abbreviations: AAD: antiarrhythmic drug, CA: catheter ablation, REF: reference group.
